# Uterine Epithelial Cell Regulation of DC-SIGN Expression Inhibits Transmitted/Founder HIV-1 *Trans* Infection by Immature Dendritic Cells

**DOI:** 10.1371/journal.pone.0014306

**Published:** 2010-12-14

**Authors:** Daniel O. Ochiel, Christina Ochsenbauer, John C. Kappes, Mimi Ghosh, John V. Fahey, Charles R. Wira

**Affiliations:** 1 Department of Physiology, Dartmouth Medical School, Lebanon, New Hampshire, United States of America; 2 Department of Medicine, University of Alabama, Birmingham, Alabama, United States of America; 3 Department of Microbiology, University of Alabama, Birmingham, Alabama, United States of America; 4 Department of Pathology, University of Alabama, Birmingham, Alabama, United States of America; University Hospital Zurich, Switzerland

## Abstract

**Background:**

Sexual transmission accounts for the majority of HIV-1 infections. In over 75% of cases, infection is initiated by a single variant (transmitted/founder virus). However, the determinants of virus selection during transmission are unknown. Host cell-cell interactions in the mucosa may be critical in regulating susceptibility to infection. We hypothesized in this study that specific immune modulators secreted by uterine epithelial cells modulate susceptibility of dendritic cells (DC) to infection with HIV-1.

**Methodology/Principal Findings:**

Here we report that uterine epithelial cell secretions (i.e. conditioned medium, CM) decreased DC-SIGN expression on immature dendritic cells *via* a transforming growth factor beta (TGF-β) mechanism. Further, CM inhibited dendritic cell-mediated *trans* infection of HIV-1 expressing envelope proteins of prototypic reference. Similarly, CM inhibited *trans* infection of HIV-1 constructs expressing envelopes of transmitted/founder viruses, variants that are selected during sexual transmission. In contrast, whereas recombinant TGF- β1 inhibited *trans* infection of prototypic reference HIV-1 by dendritic cells, TGF-β1 had a minimal effect on *trans* infection of transmitted/founder variants irrespective of the reporter system used to measure *trans* infection.

**Conclusions/Significance:**

Our results provide the first direct evidence for uterine epithelial cell regulation of dendritic cell transmission of infection with reference and transmitted/founder HIV-1 variants. These findings have immediate implications for designing strategies to prevent sexual transmission of HIV-1.

## Introduction

Since it was initially reported over 25 years ago, HIV-1 infection has remained the most challenging health issue [1]. Whereas tangible progress has been made with regards to treatment and prevention efforts [2], there is little knowledge about the biology of sexual transmission, the predominant mode of HIV-1 acquisition [3], [4]. This presents a major hurdle for strategies to reduce mucosal transmission of HIV-1, such as microbicides and vaccines. Therefore, defining the determinants of mucosal transmission remains a critical priority to the prevention of sexual acquisition of HIV-1.

Sexual transmission is almost exclusively initiated by HIV-1 variants with tropism for the CCR5 co-receptor (R5 viruses) [5], [6]. The mechanisms that facilitate preferential transmission of R5 viruses are not fully understood. Further, recent studies examining sequence composition of transmitted viruses indicate that infection is initiated by a single variant in over 75% of heterosexual HIV-1 transmission [6], [7], [8], [9], [10]. The genetic and biological characteristics of these transmitted/founder variants are under investigation [6], [9], [11].

In the female reproductive tract (FRT) mucosa, a variety of immune cell types are found, many of which are possible direct targets for HIV-1 infection [12]. Target cells in the upper and lower FRT include CD4+ T lymphocytes, macrophages, dendritic cells (DC) and epithelial cells [13], [14], [15], [16], [17]. Despite their presence, questions remain to be answered with regard to the initial target cells for infection and how HIV-1 is propagated following mucosal exposure. A spectrum of soluble immune mediators, including cytokines, chemokines and growth factors, constitute a local milieu in the sub-mucosa that regulates immune target cells [18]. With regard to HIV-1 infection *via* the FRT, we and others have demonstrated that these soluble mediators, most of which are hormonally controlled, regulate the expression of receptors and co-receptors that facilitate infection with HIV-1 [15], [19], [20].

Dynamic cell-cell communication in the FRT mucosa may influence the efficiency of HIV-1 transmission. For example, we previously described the presence of lymphoid aggregates in the endometrium, within which are CD14+ cells in close proximity to epithelial cells [15], [21]. The close anatomical juxtaposition of these myeloid cells may represent a mechanism for the regulation of their function by overlying epithelial cells. Evidence for cell-cell communication *via* local secretions is in part based on our findings that secretions from primary human uterine epithelial cells (UEC) [22], [23] modulate the differentiation and maturation processes leading to the generation of tolerogenic DC [24], [25].

In previous studies, we determined the mechanisms by which uterine epithelial cell conditioned medium (CM) modulates DC function [24]. DC which were differentiated in the presence of CM (i.e. CM-DC) had reduced surface expression of CD86 and CD83. In contrast, expression of CD1a, CD80, HLA-DR, CD14 and CD163 was not altered significantly with CM-DC relative to that seen on control DC (i.e. Con-DC). Further, following activation with TLR3 ligand (poly(I:C)) or TLR4 ligand (LPS) the expression of CD80, CD86 and CD83 were decreased on CM-DC relative to Con-DC. In addition, CM-DC responded to LPS or PIC stimulation with enhanced IL-10 production. RT-PCR analysis showed that CM-DC significantly increased expressed of mRNA for indoleamine 2,3-Dioxygenase (IDO), an immune tolerance promoting enzyme. Finally, in a mixed leukocyte reaction (MLR) assay, CM-DC had significantly lower allogeneic proliferative responses when compared to Con-DC. Interestingly, uterine epithelial cell CM does not appear to induce the development of Langerhans cells from monocytes, as indicated by the lack of expression of Langerin and E-cadherin (data not shown). These initial findings underscore the critical role of cell-cell interactions in regulating immune responses in the endometrium.

Mucosal DC are believed to play an integral role in sexual transmission of HIV-1. For example, DC express a variety of attachment receptors including DC-SIGN that enable specific binding to HIV-1 envelope proteins [26], [27], [28]. The captured virus is then transferred to target cells across the virological synapse established with DC, a process referred to as *trans* infection [29], [30], [31]. In the FRT, DC-SIGN is expressed by subsets of DC in the submucosa of the human vagina and endometrium [32], [33]. Thus, mucosal DC are poised to facilitate the dissemination of virus from the mucosal portal of entry. There is increasing recognition of mucosal DC as a unique subset whose phenotype and functional characteristics reflect specific local immune microenvironments [34]. We therefore sought to define the molecular basis by which epithelial cell-DC interactions regulate HIV-1 infection of the endometrium. We hypothesized in this study that specific immune modulators secreted by uterine epithelial cells drive the differentiation of DC with altered susceptibility to HIV-1.

We report in this study that: 1) UEC secretions down-regulate DC-SIGN expression on DC *via* a TGF-β-dependent mechanism, 2) UEC suppress the *trans* infection of both reference and transmitted/founder HIV-1, and 3) Recombinant TGF- β1 exerts a differential inhibitory effect in that it inhibits *trans* infection of reference HIV-1 but has minimal inhibitory effect on *trans* infection of transmitted/founder HIV-1 variants tested here. These findings provide potential novel insight into the biology of HIV-1 transmission in the FRT and suggest a previously unidentified mechanism of DC-SIGN regulation. Whether transmitted/founder viruses use unique *trans* infection pathways that are separate and distinct from those used by reference HIV-1 remains to be determined.

## Results

### Uterine epithelial cell secretions decrease DC-SIGN on DC by a TGF-β-dependent mechanism

**Figure 1:**
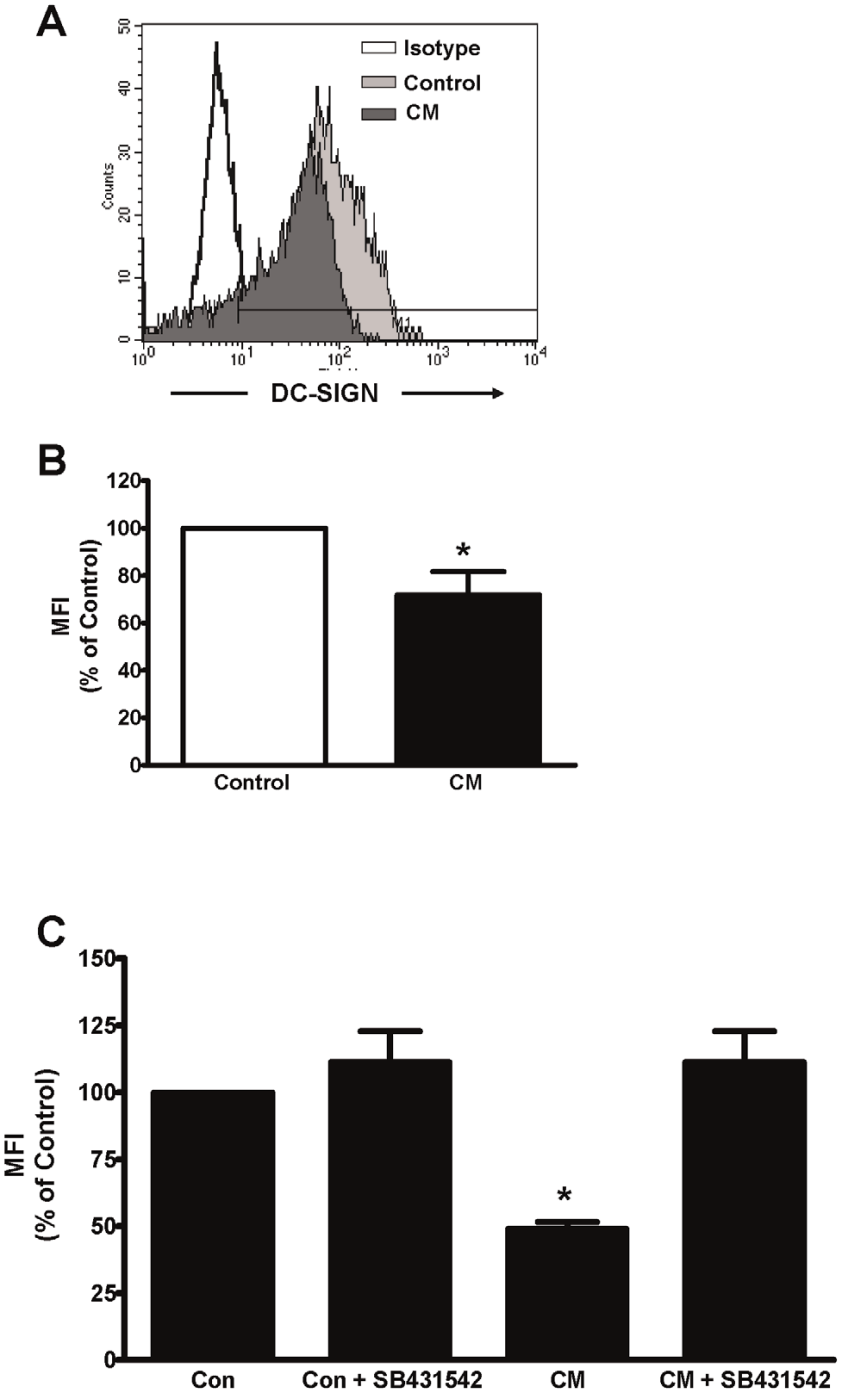
Conditioned medium (CM) from polarized uterine epithelial cells decreases DC-SIGN on Immature DC by a TGF-β mechanism. Immature DC were differentiated from human monocytes with IL-4 and GM-CSF in the presence or absence of CM obtained from polarized primary uterine epithelial cells (UEC). (**A**) Overlay histograms show expression of DC-SIGN by Control DC (light gray) or DC differentiated with CM (dark gray). Staining with isotype control antibody is shown as unfilled histogram. (**B**) Averaged mean fluorescence intensity (MFI) values for DC-SIGN expression by DC from individual donors (n = 6) are presented as % MFI of Control DC ± standard error of mean (SEM). * p = 0.035. (**C**) Blockade of TGF-β signaling abolishes the effect of CM on DC-SIGN expression. TGF-β was blocked with the TGF-β Receptor 1 kinase inhibitor (SB431542). Average MFI values for triplicate analyses from a single representative experiment (n = 3) are presented as % MFI relative to Control.

We determined the effect of primary UEC secretions on DC-SIGN expression by immature DC derived from highly purified human blood monocytes. As seen in **Figure 1A and B**, differentiation of DC for seven days in the presence of CM decreased surface expression of DC-SIGN, with a range of inhibition of 25–50% relative to Control DC (n = 6). We found a similar pattern of inhibition of DC-SIGN by CM obtained from ECC-1, a well-differentiated uterine epithelial cell line [35] (**Figure S1**). To more fully define the time interval necessary for altering DC-SIGN expression, immature DC were acutely treated with CM from ECC-1 or primary UEC for 48 hr prior to FACS analysis of DC-SIGN expression. The acute treatment with CM resulted in a similar down-regulation of DC-SIGN expression (**Figure S2**). These results demonstrate the acute and chronic effects of primary UEC CM on DC-SIGN expression.

**Figure 2:**
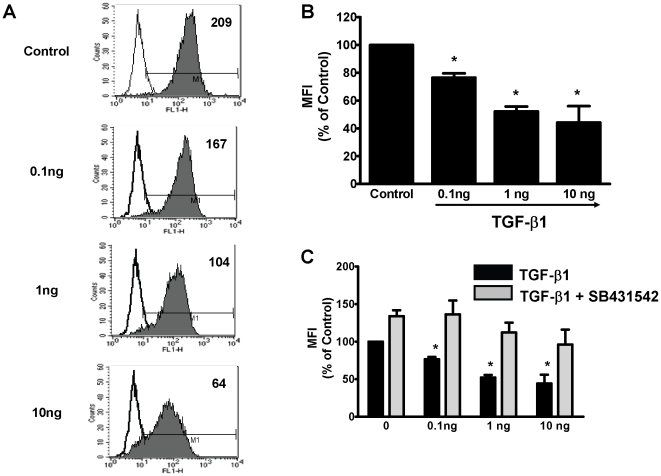
TGF-β1 inhibits DC-SIGN expression by DC. Immature DC were derived from human monocytes with IL-4 and GM-CSF in the presence or absence of increasing concentrations of recombinant TGF-β1. (**A**) Overlay histograms for DC-SIGN expression. Filled histogram shows specific staining for DC-SIGN. (**B**) Averaged mean fluorescence intensity (MFI) values for DC-SIGN expression (n = 3 donors) are shown as % MFI relative to control DC. (**C**) Expression of DC-SIGN by DC generated with TGF-β1 in the presence (black bars) or absence (gray bars) of TGF-β Receptor 1 kinase inhibitor (SB431542). * Indicates p<0.05 compared to Control DC.

We next performed experiments to identify the soluble factor in CM responsible for inhibiting DC-SIGN expression. TGF-β was evaluated as a candidate molecule due to its pivotal influence on the development and functions of DC [36]. Recognizing that polarized UEC secrete biologically active TGF- β (**Figure S3** and **Figure S4**), we assessed the role of TGF-β in CM-induced down-regulation of DC-SIGN. DC were differentiated from monocytes with CM in the presence or absence of a highly specific TGF-β type 1 receptor antagonist (SB431542) [37]. As seen in **Figure 1C** the inhibitory effect of CM on DC-SIGN expression was completely abrogated by SB431542. To extend these findings, DC were differentiated from monocytes for 7 days in the presence or absence of increasing concentrations (0, 0.1, 1 and 10 ng/mL) of recombinant human TGF-β1. TGF-β1 decreased the surface expression of DC-SIGN on immature DC in a dose-dependent manner (**Figure 2A and 2B**). Further, when DC were differentiated with TGF-β1 in presence of the TGF-β receptor 1 kinase inhibitor, SB431542 (10 µM), the inhibitory effects of TGF-β1 on DC-SIGN were blocked at all concentrations analyzed (**Figure 2C**). Taken together, these results demonstrate that biologically active TGF-β is present in uterine epithelial cell CM, and that blockade of TGF-β signaling abrogates the inhibitory effect of CM on DC-SIGN expression.

In other experiments, we determined the effect of CM obtained from either polarized primary UEC or from ECC-1 on the expression of CXCR4 and CCR5 by immature DC. For these experiments, DC were differentiated for 7 days from monocytes in the presence or absence of UEC CM. CM from primary UEC increased the expression of CXCR4 on DC (p<0.05, n = 4 donors) (**Figure S5 A**). In contrast, whereas there was a trend towards increased CCR5 expression on DC generated with CM, the effect was not statistically significant upon analysis of DC from multiple donors (p = 0.062, n = 4) (**Figure S5 B**). These findings indicate that surface expression of HIV-1 co-receptor CXCR4 but not CCR5 is enhanced by UEC derived secretions.

### Epithelial Cell CM inhibits *trans* infection of HIV-1 reference strains and transmitted/founder HIV-1 variants

**Figure 3:**
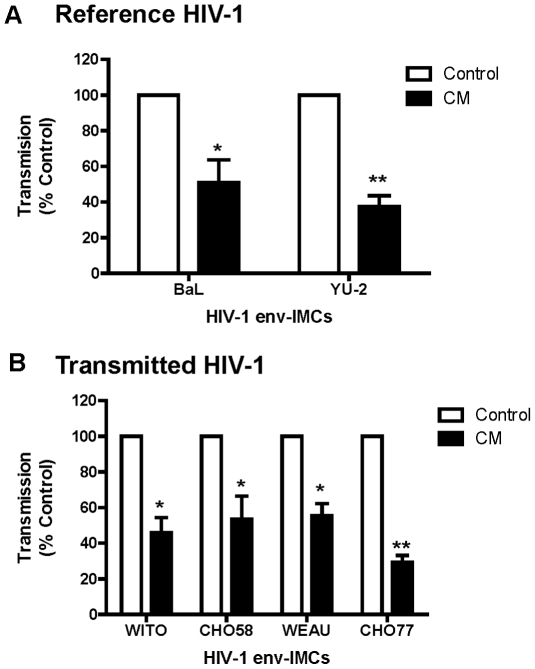
Reduced *trans* infection of HIV-1 by DC cultured with primary UEC CM. Shown is the effect of primary UEC CM on *trans* infection by DC of HIV-1 infectious molecular clones encoding envelope ectodomains (env-IMC) of reference HIV-1 (BaL or YU-2) (**A**) or transmitted/founder variants (**B**) (n = 5 blood donors). HIV-1 *trans* infection assay was performed using TZM-bl reporter cells as targets as described in materials and methods. Unshaded histograms are Control DC and the black histograms are CM DC. The data are presented as % transmission ± SEM (Calculated from 4 or 5 separate experiments: see **Table 1**) of HIV-1 by CM DC relative to Control DC. * p<0.05, ** p<0.001.

Our results showing the inhibitory effect of CM on DC-SIGN expression implied that DC generated in the presence of CM (i.e. CM-DC) might have reduced capacity for *trans* infection of HIV-1 relative to control DC (i.e. Con-DC). To directly test this hypothesis, we performed HIV-1 *trans* infection assays using a modification of a previously described protocol [38]. Immature DC were pulsed with HIV-1 derived from infectious molecular clones (IMC) expressing 6 different *env* genes, including transmitted/founder *envs* [6], [9], in an isogenic reporter backbone [39], [40] (Ochsenbauer et al, in preparation). Virus-pulsed DC were then co-cultured with TZM-bl reporter cell line [41]. Virus infection of TZM-bl cells was then assayed by measuring β-galactosidase expression. Averaged data from five experiments are shown in **Figure 3**. CM inhibited DC *trans* infection of HIV-1 expressing the envelope proteins of the reference viruses (BaL and YU-2) (**Figure 3A**). To ensure that only *trans* infection was being measured, controls carried out with each experiment included a) the lack of infection of DC when the cells were incubated with virus alone (*cis* infection) (**Figure S6**), b) pre-incubation of DC with T20 (fusion inhibitor) and/or Nevirapine (nonnucleoside reverse transcriptase inhibitor) for 1 hr prior to the addition of virus (**Figure S7 A**), and c) simultaneous addition of T20 and/or Nevirapine during co-culture of virus pulsed DC and TZM-bl (**Figure S7B**). The lack of productive infection of DC with HIV-1 coupled with the findings that *trans* infection of HIV-1 to TZM-bl was not significantly affected by pre-incubation of DC with T20 and Nevirapine or with AMD-3100 and TAK-779 prior to infection, collectively excluded productive infection of DC as a significant source of virus for *trans* infection.

**Table 1:** Effect of CM on DC-mediated *trans* infection of HIV-1.

		% Transmission (*trans*) of HIV-1 by CM DC(Transmission by Control DC set at 100%)				
		Donor 1	Donor 2	Donor 3	Donor 4	Donor 5
**Reference HIV-1**						
BaL		**61.7** (4.7)	**11.9** (2.7)	**31.0** (4.8)	**74.7** (1.0)	**75.7** (4.7)
	***p*** ** value**	0.007	0.006	0.004	0.022	0.046
YU-2		**47.8** (3.4)	**21.5** (3.7)	**23.6** (4.1)	**50.2** (1.7)	**44.2** (3.4)
	***p*** ** value**	0.010	0.0120	0.012	0.041	0.019
**Transmitted/Founder HIV-1**						
WITO		**68.6** (3.5)	**32.2** (7.1)	**48.9** (4.7)	**34.0** (5.6)	ND
	***p*** ** value**	0.001	0.002	0.021	0.025	
CH058		141.5 (21.0)	**20.6** (1.0)	**55.0**(6.9)	**43.9** (1.0)	**48.3** (7.9)
	***p*** ** value**	0.213	0.032	0.016	0.002	0.024
WEAU		**73.0** (5.7)	**35.7** (3.8)	**46.3** (6.4)	**57.1** (9.0)	**65.7** (7.0)
	***p*** ** value**	0.067	0.017	0.058	0.026	0.088
CH077		**37.2** (6.4)	**27.3** (6.9)	**19.2** (4.5)	**33.2** (3.6)	ND
	***p*** ** value**	0.006	0.014	0.003	0.023	

**Summarized data from individual experiments showing the effect of CM on** ***trans*** **infection of HIV-1 by DC.** DC were derived from monocytes in the presence or absence of CM obtained from primary UEC CM following 48 hr of culture. DC-mediated *trans* infection of HIV-1 infectious molecular clones (*env*-IMC) expressing reference *env* (BaL and YU-2) or transmitted virus *env* (WITO, CH058, WEAU and CH077) respectively was determined using TZM-bl cells as targets for virus infection. Results are presented as percent transmission ± SEM of HIV-1 by CM DC relative to Control DC (value set at 100%). ND, indicates not determined.

To determine whether CM inhibited DC *trans* infection of transmitted/founder viruses, a series of *trans* infection experiments were carried out with HIV-1 expressing envelope proteins of four different transmitted/founder variants: WITO, CH058, WEAU and CH077. As seen in **Figure 3B**, and similar to that seen with reference viruses (**Figure 3A**), CM from primary UEC decreased *trans* infection of the transmitted/founder variants. These findings were reproducible using DC from 5 donors in 5 separate experiments (**Table 1**). With the exception of one transmitted virus (CH058) which showed no inhibition in a single experiment, inhibition ranged from 27–80%.

### Transmitted HIV-1 variants are less susceptible to TGF-β1 inhibition of *trans* infection by DC

**Figure 4:**
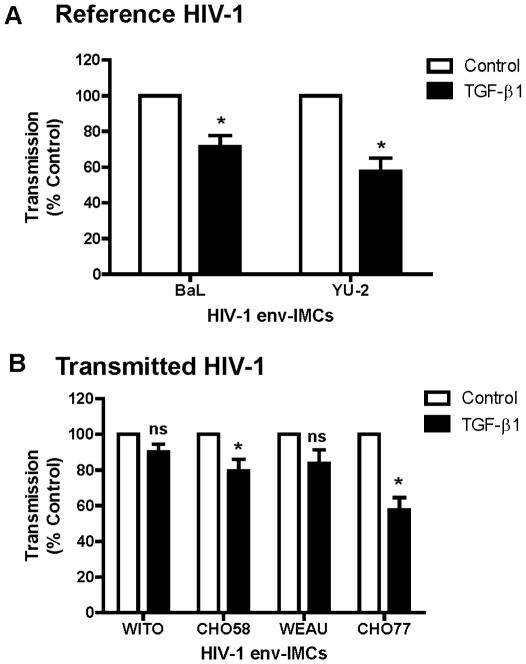
Lack of TGF- β1 inhibition of DC-mediated *trans* infection of transmitted/founder HIV-1 variants. Shown is the effect of recombinant TGF-β1 (10 ng/mL) on *trans* infection of HIV-1 infectious molecular clones (env-IMC) envelope ectodomains reference env (BaL or YU-2) (**A**) or transmitted/founder *env* variants (**B**) by DC (n = 5 blood donors). HIV-1 *trans* infection assay was performed using TZM-bl reporter cells as targets as described in materials and methods. Unshaded histograms are Control DC and the black histograms are TGF-β1 DC. The data are presented as % transmission ± SEM (Calculated from 4 or 5 separate experiments: see **Table 2**) of HIV-1 by TGF-β1 DC relative to Control DC. * p<0.05. n.s indicates not significant (p>0.05).

Having shown that TGF-β1 inhibited DC-SIGN expression by immature DC, we sought to determine its role in DC-mediated *trans* infection of HIV-1. For these experiments, DC were differentiated from monocytes in the presence or absence of recombinant TGF-β1 (10 ng/mL). This concentration corresponds to peak production of TGF-β1 by epithelial cells in the endometrium [42]. We found that DC generated in the presence of TGF-β1, when incubated with TZM-bl cells, had reduced *trans* infection of molecular HIV-1 clones expressing envelopes of reference variants (BaL and YU-2) relative to that seen with Control DC (n = 5) (**Figure 4A**). In 5 experiments with BaL and YU-2 *env*, inhibition (% Inhibition = 100%-% transmission) ranged from 12–69% and as indicated for each experiment, all TGF-β1 treated cells were significantly different from controls (*p = *0.01–0.04) (**Table 2**). Unexpectedly, we found that in contrast to HIV-1 expressing BaL and YU-2 envelopes, clones expressing envelopes from WITO, CH058 and WEAU were relatively refractory to the inhibitory effect of TGF-β1 on *trans* infection by DC (**Figure 4B**). Whereas inhibition by TGF-β1 ranged from <1 to 43%, only 4 out of 15 values from 5 experiments with transmitted variants significantly lower than those from controls (**Table 2**). The only exception was CH077 whose *trans* infection was consistently suppressed when DC were treated with TGF-β1 (4 out of 4 experiments) (**Figure 4B** and **Table 2**).

**Table 2:** Effect of Recombinant TGF-β1 on DC-mediated *trans* infection of HIV-1.

		% Transmission (*trans*) of HIV-1 by TGF-β1 DC(Transmission by Control DC set at 100%)				
		Donor 1	Donor 2	Donor 3	Donor 4	Donor 5
**Reference HIV-1**						
BaL		**51.1** (5.1)	**79.3** (2.7)	**74.0** (3.1)	**64.9** (9.0)	**87.8** (1.1)
	***p*** ** value**	0.019	0.033	0.045	0.010	0.021
YU-2		**57.6** (3.4)	**60.0** (4.9)	**72.0** (11.7)	**30.8** (7.5)	**68.6** (15.0)
	***p*** ** value**	0.010	0.042	0.039	0.026	0.012
**Transmitted/Founder HIV-1**						
WITO		**80.3** (8.1)	**100.5** (20.1)	**86.7** (9.5)	**105.7** (20.5)	**84.8** (6.5)
	***p*** ** value**	0.089	0.478	0.216	0.410	0.026
CH058		**69.3** (7.4)	**120.7** (34.0)	**90.4** (5.2)	**65.6** (19.3)	**72.1** (4.9)
	***p*** ** value**	0.044	0.300	0.178	0.147	0.011
WEAU		**78.6** (8.3)	**107.7** (6.4)	**91.6** (10.6)	**92.3** (7.4)	**56.8** (4.3)
	***p*** ** value**	0.067	0.195	0.382	0.171	0.017
CH077		**50.5** (5.9)	**68.7** (2.1)	**41.0** (1.3)	ND	**69.8** (7.8)
	***P*** ** value**	0.019	0.010	0.013		0.045

**Summarized data from individual experiments showing the effect of TGF-β1 on** ***trans*** **infection of HIV-1 by DC.** DC were differentiated in the presence or absence of TGF-β1 (10 ng/mL). DC-mediated *trans* infection of HIV-1 infectious molecular clones (*env*-IMC) expressing reference *env* (BaL and YU-2) or transmitted virus *env* (WITO, CH058, WEAU and CH077) respectively was determined using TZM-bl cells as targets for virus infection. Results are presented as percent transmission ± SEM of HIV-1 by TGF-β1 DC relative to Control DC (value set at 100%). ND, indicates not determined.

To more fully understand the mechanisms of CM and TGF-β1 mediated *trans* infection, additional experiments were conducted using a well-established T cell line, NOMI [43], [44]. Our goals in these studies were: 1) to confirm the effects CM and TGF-β1 on HIV-1 *trans* by DC, and 2) to determine the effect of antibody blockade of DC-SIGN on *trans* infection of HIV-1 expressing either reference or transmitted/founder *envs*. For these experiments, DC were pulsed with HIV-1 reporter viruses (env-IMC-lucR) expressing the *Renilla* luciferase reporter gene, and then co-cultured with NOMI cells for 72 hr. Virus replication was determined by measuring *Renilla* luciferase (LucR) activity.

**Figure 5:**
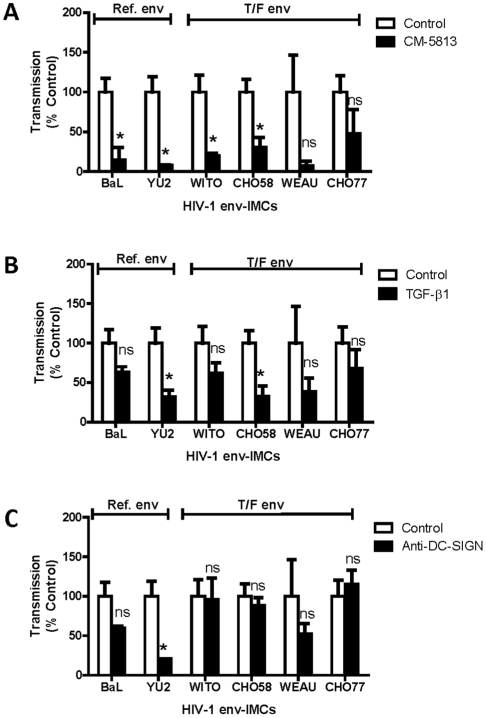
Effect of Primary UEC CM, TGF-β1 and anti-DC-SIGN on HIV-1 *trans* infection assessed with NOMI T cell targets. The effect of CM (**A**) and TGF-β1 (10 ng/mL) (**B**) on *trans* infection of HIV-1 infectious molecular clones (*env*-IMC) envelope ectodomains reference *env* (BaL or YU-2) (i.e. Ref env) or transmitted/founder *env* variants (i.e. T/F env) is shown for a representative of two experiments. (**C**) shows the effect of anti-DC-SIGN monoclonal antibody (25 µg/mL) on *trans* infection of IMC expressing Ref env and T/F env (a representative of 3 experiments). Experimental details are described in materials section. Data are presented as % HIV-1 transmission ± SEM (for triplicate analyses) relative to Control DC. * p<0.05, ns indicates non significance.

When NOMI T cells were used as targets, CM-DC had decreased capacity relative to Con-DC for *trans* infection of virus expressing BaL and YU-2 *envs* (**Figure 5A**). These results were consistent with those initially obtained with the TZM-bl system (**Figure 3A**). Further experiments conducted with the NOMI reporter system showed that CM decreased *trans* infection of virus expressing WITO and CHO58 transmitted/founder *envs* (**Figure 5A**). Unexpectedly, we found that CM had no effect on *trans* infection of virus expressing WEAU and CHO77 transmitted/founder *envs* (**Figure 5A**). This latter finding differed from that obtained with TZM-bl cells as targets (**Figure 3B**).

TGF-β1 treatment of DC showed a slightly different profile on HIV-1 *trans* infection when NOMI cells were used as targets relative to that seen with TZM-bl as targets. In contrast to the earlier observed inhibition of virus expressing reference BaL and YU-2 *envs* by TGF-β1 when TZM-bl cells were used as targets (**Figure 4A**), only YU-2 was significantly inhibited by TGF-β1 when NOMI cells were used to measure virus infection (**Figure 5B**). Of the four transmitted/founder viruses analyzed, only the *trans* infection of CHO58 was inhibited by TGF-β1 treatment of DC **(** **Figure 5B**), a result consistent with that obtained when TZM-bl cells were used as targets **(** **Figure 4B**). An expected finding was the lack of TGF-β1 inhibition of CHO77 with NOMI cells as targets. However, the lack of inhibition of *trans* infection of virus expressing WEAU and WITO *env* by DC treated with TGF-β1 (**Figure 5B**) was consistent with the results initially obtained with the TZM-bl reporter system (**Figure 4B**).

Having demonstrated that the expression of DC-SIGN on DC is decreased by CM (**Figure 1**) and TGF-β1 (**Figure 2**), we examined the effect of DC-SIGN blockade on *trans* infection of viruses expressing reference and transmitted/founder *envs*. For these experiments, DC were pre-pretreated with antibody to DC-SIGN prior to incubation with HIV-1. As seen in Figure 5, blockade of DC-SIGN decreased *trans* infection of HIV-1 IMC expressing reference YU-2 *env*, but had little to no significant effect on *trans* infection of IMC expressing reference BaL *env* (or virus expressing WITO, CH58, WEAU and CHO77 transmitted/founder *envs* (**Figure 5C**).

## Discussion

DC present in the mucosal tissues of the FRT may be integral to the cellular and molecular processes that underlie immune protection and possibly contribute to the sexual transmission of HIV-1. Within the mucosa, the phenotype and functions of DC are influenced by specific regulatory cues in the local milieu. The aim of our study was to elucidate the molecular mechanisms by which endometrial epithelial cells modulate HIV-1 infectivity of DC. We report that primary UEC CM decreases the expression of DC-SIGN on DC *via* a TGF-β-dependent mechanism. Further, DC generated with epithelial secretions exhibited reduced *trans* infection of HIV-1 expressing envelope proteins of reference (BaL and YU-2) and of four transmitted/founder HIV-1 (WITO, CH058, WEAU and CH077). In contrast, recombinant TGF-β1 inhibited DC-mediated *trans* infection of reference viruses but failed to consistently inhibit *trans* infection of transmitted/founder variants. Collectively, our findings provide unique and novel insight into the nature of cell-cell interactions in the endometrial mucosa, as well as the potential impact of such interactions on HIV-1 capture and dissemination by DC within the FRT.

Our study shows that secretions from primary UEC down-regulate the expression of DC-SIGN by immature DC. The results we obtained with TGF-β receptor blockade and with recombinant TGF-β1 support the hypothesis that the effects of primary UEC secretions on DC-SIGN are at least in part attributable to TGF-β. Whereas TGF-β has been previously shown to decrease the expression of DC-SIGN on DC [45], [46], our study is the first to directly link TGF-β derived from polarized uterine epithelial cells with the regulation of DC-SIGN expression by DC. TGF-β is known to be secreted in both an activated form and as an inactive molecule bound to latency associated protein (LAP) [46]. Others have shown that primary UEC secrete TGF-β [42], [47]. Our findings extend these studies, by using a highly sensitive and specific bioassay [48], to demonstrate that primary UEC secrete biologically active TGF-β. Overall, our findings demonstrate that epithelial cells from the endometrium secrete bioactive TGF-β, which exerts an inhibitory effect on immature DC during their differentiation from myeloid precursors to lower surface expression of DC-SIGN.

Our results indicate that CM from primary uterine epithelial cells inhibits *trans* infection of HIV-1 by DC. Using the TZM-bl reporter cell line as target, we found that CM inhibited *trans* infection of prototypic R5 reference viruses (BaL and YU-2) and transmitted/founder variants (WITO, CH058, CH077 and WEAU). Similarly, in an alternative approach that utilizes CD4+/CCR5+ T cells, the NOMI reporter cell line [43], [44] as target, CM inhibited *trans* infection of reference viruses and two of the transmitted viruses (WITO and CHO58). Unexpectedly, *trans* infection of WEAU and CHO77 was not inhibited by CM when NOMI cells were used targets. The mechanisms that underlie this observation are uncertain, and may involve both inherent properties of the viruses as well as disparate specific mechanisms by which DC transfer HIV-1 to TZM-bl or T cells. Nevertheless, that transmitted HIV viruses were inhibited, as were reference strains, is of particular importance given the new paradigm that sexual transmission of HIV-1 is most likely a single variant in over 75% of transmissions [6], [7], [8], [9], [10]. Epidemiological studies have shown that the rate of male-to-female transmission of HIV-1 is relatively low compared to other sexually transmitted infections (STIs) [49], [50]. While the reasons for this low rate of HIV mucosal transmission are likely to be multi-factorial, our findings suggest that soluble immune mediators produced by epithelial cells reduce the capacity of DC to disseminate HIV-1 in the endometrium. The recognition that dyes and semen placed in the vagina are rapidly distributed throughout the FRT [51], [52], [53] is supportive of the evidence that both cell-free and cell-associated HIV-1 enters the lumen of the cervix, uterus and the Fallopian tube during a window of vulnerability [54]. Our studies suggest that during male-to-female transmission, secretions derived from epithelial cells of the FRT act to reduce the overall efficiency of HIV-1 transmission by inhibiting DC-mediated *trans* infection.

In contrast to our findings with CM, TGF-β1 inhibited DC-mediated *trans* infection of the reference R5 HIV-1 strains (BaL and YU-2), but failed to consistently inhibit *trans* infection of transmitted/founder variants. These studies indicate that epithelial cell secretions (CM) are able to inhibit transmitted viruses in ways beyond that seen with TGF-β1 alone. Whether the differences observed between reference and transmitted viruses are due to inherent binding affinities of the viruses for DC-SIGN remain to be determined. However, by analyzing the effects of TGF-β1, the present studies have unmasked a level of complexity in endometrial epithelial cell and DC function not previously recognized. Specifically, the failure of TGF-β1 to inhibit *trans* infection of transmitted/founder viruses suggests that viral *trans* infection can occur independently of DC-SIGN. One possible explanation for these observations is that several isoforms of TGF-β1, TGF-β2 and TGF-β3 [47] are present in uterine epithelial cell CM. It is possible that synergistic interactions between these isoforms confer primary UEC CM with a broader inhibitory activity on DC-mediated *trans* infection of HIV-1.

We have previously characterized the spectrum of soluble factors produced by polarized uterine epithelial cells in culture, and found that in addition to TGF-β, these cells secrete a number of cytokines and chemokines [22], [23], [55], [56], [57]. Some of these factors, including human defensin 2 (HBD2), G-CSF, M-CSF, TSLP and hepatocyte growth factor (HGF), are known to directly affect the differentiation and/or activation of DC [58], [59], [60], [61], [62], [63]. It is therefore likely that a number of soluble factors produced by uterine epithelial cells, in addition to TGF-β, interact in a specific manner to exert the observed inhibitory effect on HIV-1 *trans* infection.

An additional candidate molecule that could mediate the effects of uterine epithelial cell CM on HIV-1 *trans* infection is Activin A. As a member of the TGFβ superfamily [64], Activin A is produced by human uterine epithelial cells [65], [66], [67]. A recent study showed that Activin A induces the differentiation of LCs from human monocytes [68]. In preliminary experiments, we examined the effect of Activin A on HIV-1 *trans* infection by DC. We found that DC differentiated in the presence of recombinant Activin-A (i.e. Activin-DC) had reduced capacity for *trans* of IMC expressing envelopes of BaL *env* and YU-2 *env*-expressing reference viruses (**Figure S8**). Similarly, Activin-DC displayed reduced *trans* infection of two IMC expressing envelopes of transmitted/founder viruses, WITO and CH58 (**Figure S8**). Thus, in addition to TGF-β, Activin A activity may account for the broader inhibitory effects of uterine epithelial cell CM on HIV-1 *trans* infection mediated by DC. Future studies examining the mechanisms by which Activin A, either singly or in synergy with TGF-β affect HIV-1 *trans* infection will provide valuable insight about biology of sexual transmission.

An alternative explanation for the observed minimal inhibitory effects of TGF-β1 on *trans* infection of transmitted/founder viruses could be the involvement of DC-SIGN-independent pathways including the use of alternate HIV-1 capture/binding molecules such as galactosyl ceramide (Gal-ceramide), DEC-205, DCIR [69], [70], [71], [72], [73], [74], [75]. In our study, specific blockade of DC-SIGN on DC had little or no effect on the *trans* infection of transmitted/founder HIV-1 variants, suggesting the involvement of alternate receptors in addition to DC-SIGN. That infection is inhibited by epithelial cell CM suggests that secretions from epithelial cells have the ability to inhibit the alternate capture molecules utilized by transmitted/founder viruses.

Our findings indicate that epithelial cell CM inhibits *trans* infection of HIV-1 constructs expressing envelopes of transmitted/founder viruses more effectively than does TGF-β1. In other studies, we obtained evidence of potentially important phenotypic differences between commonly used ‘reference virus strains’ like BaL and YU-2, and transmitted/founder HIV-1 variants. For example, while Clade B transmitted/founder Envs utilize CCR5 as a co-receptor [6], we found that a panel of ten Clade B transmitted/founder HIV-1 IMC, which we recently generated, replicate poorly in monocyte-derived macrophages, and that this maps to *env* (Ochsenbauer et al., unpublished observations). A similar phenotype was observed for 3 Clade C transmitted/founder IMC [9]. In contrast, the reference viruses BaL and YU-2 are highly macrophage-tropic. We do not infer that a lack of macrophage tropism is the cause for the phenotype observed here, but rather intend to highlight that certain phenotypic differences between transmitted/founder viruses and commonly used reference viruses may and do exist, and that it is therefore important to include the most relevant virus variants in transmission-related studies. Our findings in the present study suggest that receptors on DC through which reference and transmitted/founder HIV-1 *trans* infection occurs may be separate and distinct. However, too little is presently known about the biology of transmitted/founder HIV-1 envelopes to propose a mechanistic basis for our findings.

In conclusion, our study reports a novel mechanism by which epithelial cells regulate HIV-1 infectivity in the endometrium. The recognition that uterine epithelial cell secretions interfere with DC-mediated *trans* infection of HIV-1 in the endometrium offers new insight into potential novel strategies to block sexual transmission of HIV-1 transmitted/founder viruses.

## Materials and Methods

### Isolation and Culture of Uterine Epithelial Cells (UEC)

All investigations involving human subjects were conducted according to the principles expressed in the Declaration of Helsinki. Approval to use human endometrial tissues in this study was previously obtained from the Committee for the Protection of Human Subjects, Dartmouth Hitchcock Medical Center (DHMC), and written informed consent was obtained from the patients before surgery. Primary UEC were isolated from endometrial tissues of patients undergoing hysterectomy at DHMC. Primary UEC were then cultured to establish polarity with apical and basolateral compartments as previously described [55]. Briefly, following enzymatic digestion to isolate epithelial cells from uterine tissues, cells were washed, resuspended in complete DMEM/F12 medium without phenol red (Invitrogen Life Technologies, Carlsbad, CA) supplemented with 20 mM HEPES, 2 mM L-glutamine (Invitrogen Life Technologies, Carlsbad, CA), 50 µg/mL primocin (Invivogen, San Diego, CA, USA) and 10% heat-inactivated defined Fetal Bovine Serum (FBS) (Hyclone, Logan, UT, USA). Cells were then analyzed for cell number and viability. On average, isolated primary UEC are cultured on inserts for two weeks to ensure tight junction formation, which typically occurs in 7–10 days [76]. Upon attainment of high TER, the apical and basolateral compartments were washed 4 times with complete DMEM/F12 medium. Cells with high TER were further cultured in complete DMEM/F12 medium for 24 hr prior to the collection of apical and basolateral conditioned medium (CM). The collected media were centrifuged for 5 min at 10, 000× g and stored at −80°C. Basolateral CM harvested from polarized primary UEC after 24 hr of culture was used in all DC cultures. Apical and basolateral CM was collected from the confluent monolayers of UEC after 24 hr in culture, and used in subsequent studies.

ECC-1, a well-differentiated uterine epithelial cell line [35] was a gift from Dr George Olt, Penn State College of Medicine, Milton S. Hershey Medical Center, PA. ECC-1 were cultured as previously described to establish cellular polarity with apical and basolateral compartments [22]. CM was collected from polarized ECC-1 following 24 hr of culture and used in subsequent studies.

### Isolation of Monocytes and Preparation of dendritic cells (DC)

Immature DC were differentiated from human monocytes with GM-CSF and IL-4 [77]. Briefly, monocytes were isolated from human PBMC using CD14 microbeads (Miltenyi Biotec, Auburn, CA). The purity of isolated monocytes was greater than 98% as indicated by CD14 expression (anti-CD14 mAb, clone 61D3, Ebioscience, San Diego, CA). Isolated monocytes were subsequently were cultured for 7 days with complete RPMI 1640 medium without phenol red (Invitrogen Life Technologies) supplemented with 20 mM HEPES, 2 mM L-glutamine (Invitrogen Life Technologies), 50 µg/mL primocin (Invivogen, San Diego, CA, USA) and 10% heat-inactivated defined FBS (Hyclone). On day 0 of culture, cells were treated with GM-CSF (50 ng/ml) and IL-4 (50 ng/ml) (PeproTech Inc). Thereafter, cytokines were replenished every other day (i.e. days 2, 4 and 6). On day 7 immature DC were harvested, counted and viable cells resuspended for use in subsequent experiments.

To determine the effect of UEC CM on differentiation of immature DC, human monocytes were cultured with IL-4 (50 ng/mL) and GM-CSF (50 ng/mL) (PeproTech Inc) for seven days in the presence or absence of CM obtained from ECC-1 or from primary UEC. In all experiments, CM from ECC-1 and primary UEC were used at 1∶1 dilution with fresh complete RPMI medium to avoid nutritional deprivation to the cells during the differentiation process. In some experiments, TGF-β Receptor 1 blocker, SB431542 (10 µM, Tocris Cookson Inc) [37] was simultaneously added with CM for the entire duration of the differentiation. As positive control for TGF-β effects, DC were differentiated with increasing concentrations (0, 0.1 ng, 1 ng and 10 ng/mL) of recombinant human TGF-β1 (PeproTech Inc).

### Flow cytometry

Immature DC were stained with the following flourochrome-labeled antibodies: DC-SIGN (Clone 120507), CCR5 (Clone 45531) and CXCR4 (Clone 12G5) (R&D (Minneapolis, MN). Matched isotype controls for the antibodies were used to control for non-specific binding. Following antibody staining, cells were washed with staining buffer and fixed with 2% methanol-free paraformaldehyde in 1X PBS (2% PFA), and then subsequently analyzed on a Becton Dickinson FACS Calibur (San Jose, CA). The acquired FACS data were analyzed with the Cell Quest™ software (BD).

### TGF-β ELISA and Bioassay

TGF-β1 concentrations in the CM were analyzed with the DuoSet ELISA kit (R&D Systems, Minneapolis, MN) according to the manufacturer's recommendations. The biological activity of TGF-β in the CM was determined with the Mv1Lu mink lung epithelial cell line as previously described [48].

### HIV-1 *trans* infection assay

Viruses used were replication-competent infectious molecular clones (IMC) of a modified NL4-3 backbone into which the *env* sequences of primary HIV-1 isolates were cloned (*env*-IMC) including those of transmitted viruses [6], [9] (Ochsenbauer et al, in preparation). The *nef*-positive *gfp*-encoding NLENGIi-Env.ecto viruses [39], [40] and (Ochsenbauer et al in preparation) derived from NLENGI-ires [39], [78], [79] express GFP upon infection which was exploited to confirm the absence of DC *cis*-infection under the given experimental conditions (data not shown). NL-LucR.T2A-Env.ecto viruses express the *Renilla* luciferase reporter gene [40]. All *env*-IMC constructs expressed Env proteins in which the ectodomains were derived from different primary viruses, respectively, and the cytoplasmic domain of NL4-3 Env. The strategy and the rationale of shuttling in the Env ectodomain encoding sequences from select primary viruses into isogenic reporter and non-reporter virus backbone has been described [40], [80]. Virus was produced on 293 T cells and titer was established on TZM-bl cells as described [41]. HIV-1 *trans* infection by DC was assayed with a modification of previously described protocols [81], [82]. Following differentiation of immature DC for 7 days, cells were extensively washed and then pulsed with HIV-1 for 2 hr. Unbound virus was removed by multiple wash steps and DC then co-cultured with TZM-bl cells for 48 hr. Virus infection of TZM-bl cells was determined by measuring beta galactosidase activity following addition of Beta Glo substrate.

In some experiments, HIV-1 *trans* infection by DC was assayed with T cells as target, utilizing the previously described reporter T cell line, NOMI [43], [44]. NOMI cells were maintained in RPMI supplemented with 10% FBS. For these *trans* infection assays, DC were pulsed for 2 hr with *env*-IMC expressing *Renilla* luciferase reporter gene [40]. Following multiple wash steps to remove unbound virus, DC were co-cultured with NOMI cells for 72 hr. Virus infection of NOMI cells was then assessed by measuring *Renilla* luciferase activity using commercial reagents (Promega, Madison, WI). Additional control experiments were performed to directly examine the involvement of DC-SIGN in DC mediated *trans* infection of HIV-1. For these experiments, DC were pre-treated with anti-DC-SIGN antibody (25 µg/mL, clone 120507 from R&D) for 30 minutes at 37°C prior to 2 hr incubation with HIV-1.

### Statistical Analysis

Statistical analysis was performed using unpaired t-test. One way ANOVA followed by a Tukey multiple comparison (GraphPad Prism Version 5.0; GraphPad Software Inc., San Diego, CA) was used to determine differences between treatment conditions. A value of *p*<0.05 was considered significant.

## Supporting Information

Figure S1 ECC1 Conditioned medium (CM) decreases expression of DC-SIGN on Immature DC by TGF-β mechanism. (A) Overlay histograms show expression of DC-SIGN by Control DC (light gray) or DC differentiated with ECC-1 CM (dark gray). Staining with isotype control antibody is shown as unfilled histogram. Combined MFI values for DC-SIGN expression by DC from multiple donors (n = 5) are presented as % MFI of Control DC ± standard error of mean (SEM). * indicates p = 0.035. (B) Blockade of TGF-β signaling abolishes the effect of ECC-1 CM on DC-SIGN expression. TGF-β was blocked with the TGF-β Receptor 1 antagonist (SB431542). Combined MFI values (n = 3 donors) are presented as % MFI of Control DC ± SEM. * indicates p<0.009 compared to control DC.
(0.10 MB TIF)

Figure S2 Acute treatment of DC with uterine epithelial cell CM down-regulates DC-SIGN expression. Monocytes were cultured in IL-4 (50 ng/mL) and GM-CSF (50 ng/mL) for 7 days. Immature DC were cultured for additional 48 hr in the absence or presence of ECC-1 CM (A) or primary UEC CM (B). Results are presented as % MFI DC-SIGN expression relative to Control DC. * indicates p<0.05 (n = 3 donors).
(0.00 MB TIF)

Figure S3 TGF-β1 Secretion by Uterine Epithelial Cells. TGF-β1 was measured in basolateral and apical supernatants obtained from primary uterine epithelial cells (n = 5) following 48 hr of culture. Active TGF-β1 was measured directly while total TGF-β1 was assayed following an activation step with HCL. Data show mean ± standard error of mean (s.e.m) of TGF-β1 in either the apical or basolateral chamber of the Transwell tissue culture insert. * Indicates significant difference (p<0.05) from secretion into the apical chamber.
(0.01 MB TIF)

Figure S4 Biological activity of TGF-β produced by primary uterine epithelial cells (UEC). The activity of TGF-β was determined as previously described [39]. (A) Standard curve generated with increasing doses of recombinant TGF-β1. Shown is a dose-dependent induction of PAI-1 (plasminogen activator inhibitor-1) in Mink lungs epithelial cells (MLECs) by TGF-β1. (B) High concentration (TGF-β1, 10 ng/mL) induces PAI-1 expression in MLECs. The effect is blocked by treating the cells with SB431542 (10 µM). Included are baseline inductions of PAI-1 in MLECs by DMEM in the absence (Medium) or presence (Medium-Block) of SB431542. (C) Effect of primary UEC CM on PAI-1 (Plasminogen activator inhibitor 1) expression. MLECs were cultured overnight in the presence (UEC CM) or absence (Medium) of primary UEC CM (n = 5) obtained from basolateral compartment of polarized epithelial cells. The specificity of this effect was determined by treating the cells with SB431542 (UEC CM-Block). Data are presented as mean Relative light units (RLU) of luciferase activity ± standard error of mean (SEM).
(0.08 MB TIF)

Figure S5 Uterine epithelial cell conditioned medium (CM) increases surface expression of CXCR4 on Immature DC. Overlay histograms for the expression of CXCR4 (A) and CCR5 (B) by Control DC or DC differentiated with primary UEC Conditioned medium (CM) for 7 days. The unfilled histogram shows staining with matched isotype antibody. Combined MFI values for DC-SIGN expression by DC from healthy donors (n = 4) that have been differentiated in the presence (black bar) or absence (white bar) of UEC CM. Data is presented as % MFI of Control DC ± standard error of mean (sem). * p<0.05, n.s denotes not significant (p = 0.062).
(0.28 MB TIF)

Figure S6 Relatively low productive infectivity of DC with HIV-1. DC cells (2×105) were incubated with 3×105 infectious units (IU) of HIV-1 infectious molecular clones (env-IMC-LucR) expressing reference HIV-1 envelopes (BaL or YU2) (A) or envelopes of transmitted/founder variants (B) with Renilla luciferase reporter gene. For comparison, infection of TZM-bl cells with HIV-1 encoding BaL or YU-2 env is shown (C). Following 3 days of incubation, cells were lysed with 1X lysis buffer and luciferase activity was determined with commercial reagents (Promega, Madison, WI). Results are presented as mean ± SEM of luciferase activity (LucR) after blank correction (RLU of blank  = 168.60). The bold dashed line shows 2.5-fold change in luciferase activity above background which is considered significant. *p<0.05.
(0.02 MB TIF)

Figure S7 Control experiments to optimize HIV-1 trans infection assay. (A) DC were incubated for 1 hr in the presence or absence of Nevirapine (10 mM) and T20 (10 mg/mL) prior to incubation with infectious molecular clones (env-IMC) expressing envelope proteins of BaL or YU2 (5×104 IU). DC were then washed and co-cultured with TZM-bl. (B) DC were infected with BaL env or YU-2 env expressing env-IMC for 2 hr and then co-cultured with TZM-bl (2.5×105) in the presence or absence of T20 (10 mg/mL). C) DC were incubated for 1 hr in the presence or absence of AMD-3100 (1.2 mM) and TAK-779 (10 mM). The cells were washed extensively prior to infection with BaL env expressing env-IMC for 2 hr. DC were then washed and co-cultured with TZM-bl. In all cases, virus infection of TZM-bl cells was then assayed by measuring β-galactosidase expression. Results are presented as mean Relative Light Units (mean ± SEM) of β-galactosidase activity (beta-Gal RLU).
(0.01 MB TIF)

Figure S8 Effect of Activin A treatment on trans infection of HIV-1 by DC. DC were generated from human monocytes in the absence (Control DC) or presence of recombinant Activin A (100 ng/mL, ie. Activin DC). Following 7 days of differentiation, DC were pulsed with HIV-1 infectious molecular clones (env-IMC-LucR) expressing envelope ectodomains of reference HIV-1 (BaL or YU-2) (A) or transmitted/founder variants (B) for 2 hr and then co-cultured with NOMI cells for 3 days. Virus replication in NOMI cells was determined by measuring Renilla luciferase activity. Unshaded histograms are Control DC and the black histograms are Activin A treated DC. The data are presented as % transmission ± SEM (for triplicate analyses of a single experiment) of HIV-1 by Activin DC relative to Control DC.
(0.00 MB TIF)
