# Supplementary figures and images for: Uterine Epithelial Cell Regulation of DC-SIGN Expression Inhibits Transmitted/Founder HIV-1 Trans Infection by Immature Dendritic Cells
Source: PLoS One. 2010 Dec 14;5(12):e14306. doi: 10.1371/journal.pone.0014306 (PMC3001862; doi:10.1371/journal.pone.0014306)

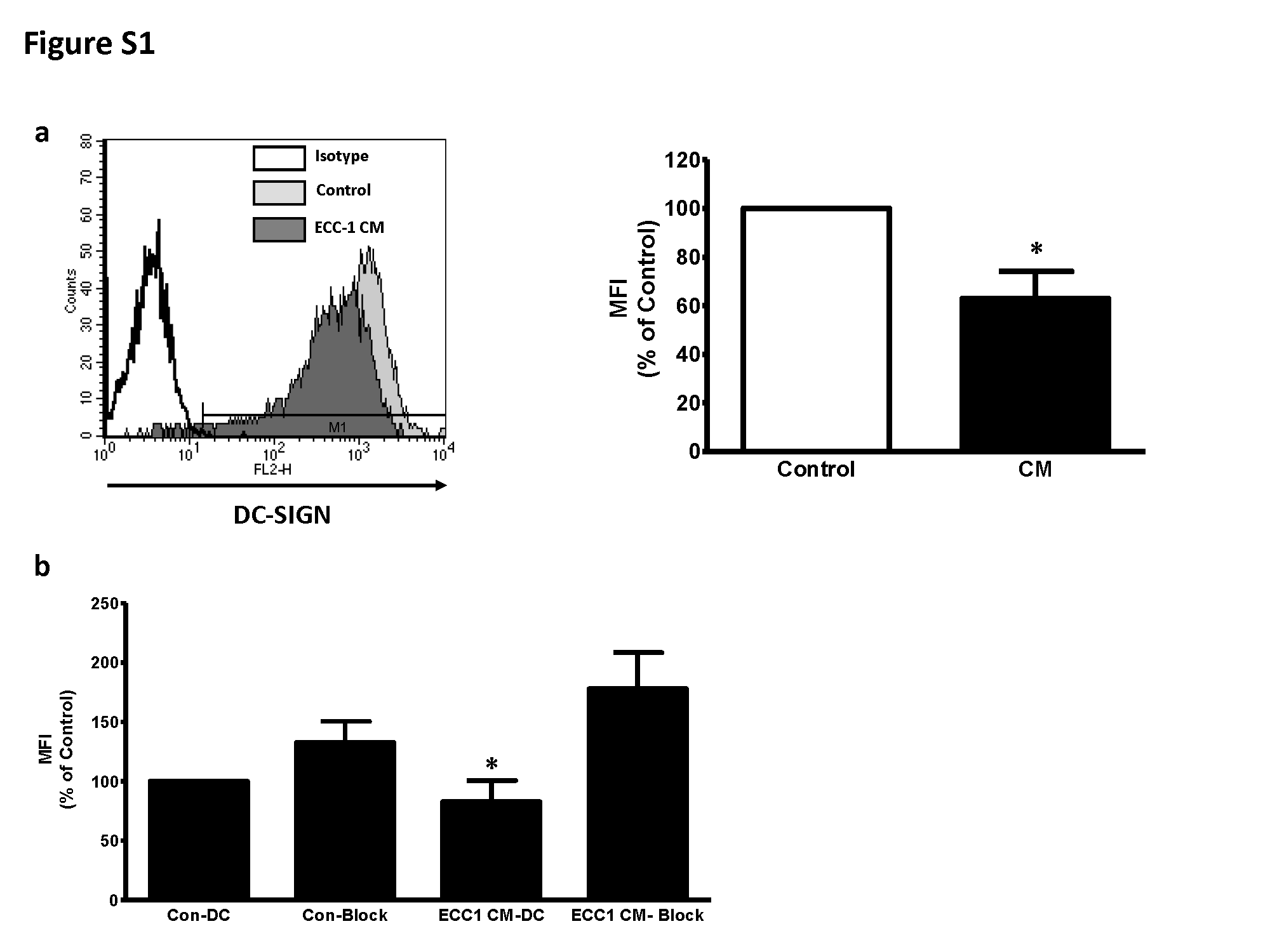

Supplement: Figure S1 — ECC1 Conditioned medium (CM) decreases expression of DC-SIGN on Immature DC by TGF-β mechanism. (A) Overlay histograms show expression of DC-SIGN by Control DC (light gray) or DC differentiated with ECC-1 CM (dark gray). Staining with isotype control antibody is shown as unfilled histogram. Combined MFI values for DC-SIGN expression by DC from multiple donors (n = 5) are presented as % MFI of Control DC ± standard error of mean (SEM). * indicates p = 0.035. (B) Blockade of TGF-β signaling abolishes the effect of ECC-1 CM on DC-SIGN expression. TGF-β was blocked with the TGF-β Receptor 1 antagonist (SB431542). Combined MFI values (n = 3 donors) are presented as % MFI of Control DC ± SEM. * indicates p<0.009 compared to control DC. (0.10 MB TIF) [file pone.0014306.s001.tif]

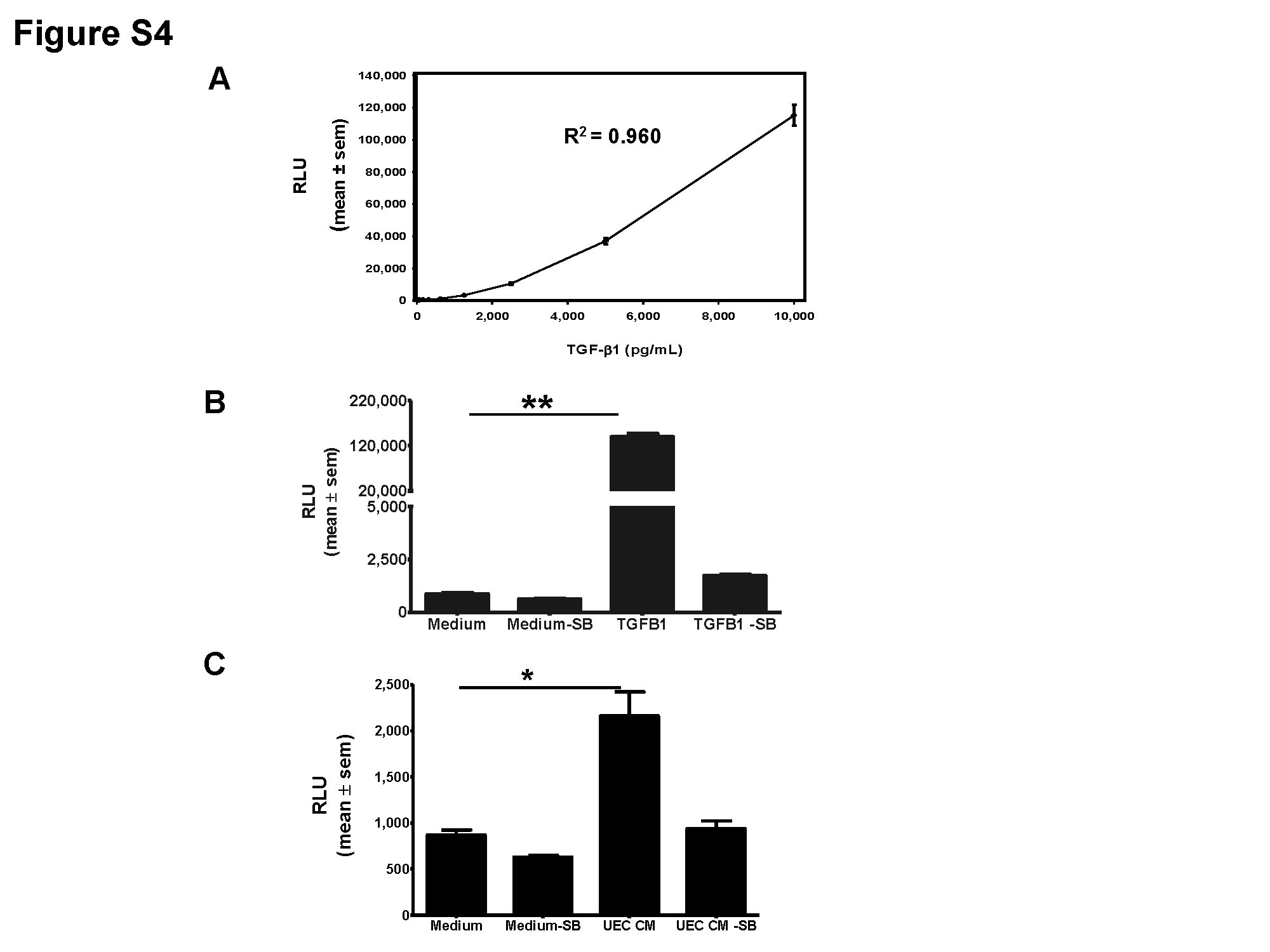

Supplement: Figure S4 — Biological activity of TGF-β produced by primary uterine epithelial cells (UEC). The activity of TGF-β was determined as previously described [39]. (A) Standard curve generated with increasing doses of recombinant TGF-β1. Shown is a dose-dependent induction of PAI-1 (plasminogen activator inhibitor-1) in Mink lungs epithelial cells (MLECs) by TGF-β1. (B) High concentration (TGF-β1, 10 ng/mL) induces PAI-1 expression in MLECs. The effect is blocked by treating the cells with SB431542 (10 µM). Included are baseline inductions of PAI-1 in MLECs by DMEM in the absence (Medium) or presence (Medium-Block) of SB431542. (C) Effect of primary UEC CM on PAI-1 (Plasminogen activator inhibitor 1) expression. MLECs were cultured overnight in the presence (UEC CM) or absence (Medium) of primary UEC CM (n = 5) obtained from basolateral compartment of polarized epithelial cells. The specificity of this effect was determined by treating the cells with SB431542 (UEC CM-Block). Data are presented as mean Relative light units (RLU) of luciferase activity ± standard error of mean (SEM). (0.08 MB TIF) [file pone.0014306.s004.tif]

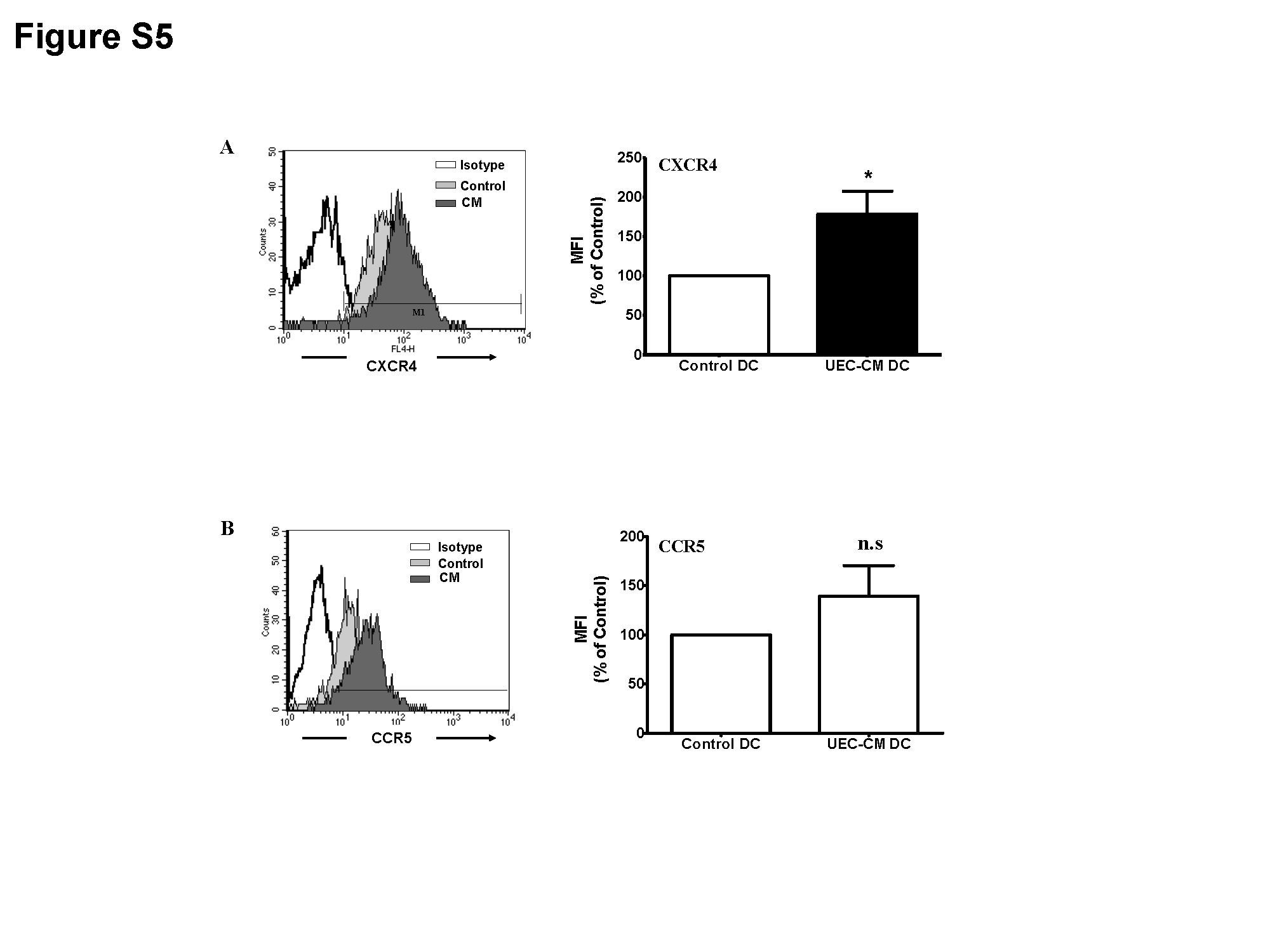

Supplement: Figure S5 — Uterine epithelial cell conditioned medium (CM) increases surface expression of CXCR4 on Immature DC. Overlay histograms for the expression of CXCR4 (A) and CCR5 (B) by Control DC or DC differentiated with primary UEC Conditioned medium (CM) for 7 days. The unfilled histogram shows staining with matched isotype antibody. Combined MFI values for DC-SIGN expression by DC from healthy donors (n = 4) that have been differentiated in the presence (black bar) or absence (white bar) of UEC CM. Data is presented as % MFI of Control DC ± standard error of mean (sem). * p<0.05, n.s denotes not significant (p = 0.062). (0.28 MB TIF) [file pone.0014306.s005.tif]
